# 

**Published:** 1869-05

**Authors:** 


### AMERICAN JOURNAL

OF

### DENTAL SCIENCE,

EDITED BY

F. J. S. GORGAS, M. D., D. D. S.

VOL. 3.—THIRD SERIES.—NO. 12.

Surgeon Genl's Office LIBRARY 39613 Washington D.C.

BALTIMORE:

SNOWDEN & COWMAN.

LONDON:

TRUBNER & CO., 60 PATERNOSTER ROW.

1870.

W
1
AM 45 M

3375

### INDEX TO VOL. III.--THIRD SERIES.

A Case of Hemorrhage following the Extraction of a Tooth. By J. D. Patterson, - - - - - - - - - 362
A Human Monstrosity, - - - - - - - - 70
A New Cement, - - - - - - - - - - - 542
A New Medical Journal. Baltimore Medical Journal, - 446
A New Mode of Administering Nitrous Oxide Gas, - - 445
A New Substitute for Phosphorus in Lucifer Matches, - 344
A Novel Case and Treatment. By S. J. Cobb, - - - 566
A Novel Tumor, - - - - - - - - - - 292
A Peculiar Form of Ulceration of the Gum, - - - - 24
A Singular Case. By James B. Hodgkin, D.D.S. - - 112
A Tooth Driven into the Antrum, - - - - - - 592
A Valuable Cement, - - - - - - - - - 438
A Young Tobacco Chewer, - - - - - - - - 388
Aconite Tincture, Poisoning by, - - - - - - 544
Action of Nitrate of Silver on Nervous Tissue, - - - 593
Adhesive Gold Foil. Its Discovery, History and Use. By A. Westcott, M.D., D.D.S. - - - - - - - - 523
Alarming Dental Hemorrhage. By A. J. Eidson, M.D., - 180
Aluminum, - - - - - - - - - - - - - 387
Alveolar Abscess. Treatment of, By C. S. Chittenden, - 181
American Academy of Dental Science. By Edward N. Harris, D.D.S., - - - - - - - - - - - - 367
American Association for the Advancement of Science, - 299
American Dental Association, - - - - - - - - 70
American Dental Association, Proceedings of, By W. C. Horne, D.D.S., - - - - - - - - - 236
American Dental Association, Letter from Committee of Arrangements of, - - - - - - - - 116
American Dental Convention, Annual Meeting of, By J. H. Smith, - - - - - - - - - - - 422
Amputation under Choral, - - - - - - - - - 537
An Ingenious Method of Drying Animal and Vegetable Substances, - - - - - - - - - - - 440
Anæsthetic Inhalation, - - - - - - - - - 597
Anæsthetics and their Administration, By D. H. Goodwillie, M.D., D.D.S., - - - - - - - - - - 71
Anæsthetics in Dental Operations, By Thomas C. Edwards, D.D.S. - - - - - - - - - - - 101
Anœsthetics for Dental Operations, By Walter Bruce, D.D.S. 307–351
Anatomy. An Introductory Lecture on, By Prof. E. Lloyd Howard, M.D., - - - - - - - - - 402

Anatomy and Physiology of the Teeth, by James B. Hodgkin, D.D.S. - - - - - - - - - - 161
Angell, Jno. G. D.D.S., - - - - - - - - 217
Another Champion in the Field, - - - - - - - 199
Antidote to Carbolic Acid, - - - - - - 147
Antrum. Disease of, - - - - - - - - - 67.573
Arkansas Stones, - - - - - - - - - - 145
Artificial Respiration, By Sumner Rhoades, M.D., - - - 434
Atmospheric Pressure over Alveolar Ridge. By W. Geo. Beers, 143
Augspath Louis, D.D.S., - - - - - - - - 567
Austen, Professor P. H. M.D., D.D.S., - - - - 399-497
Babcock, J. F., - - - - - - - - - - - 473
Baltimore College of Dental Surgery. Thirtieth Annual Commencement of, - - - - - - - - - 542
Barr, Jno. R., D.D.S., - - - - - - - - 256
Bean, Jas. B., M.D., D.D.S., - - - - - - - 151
Beers, W. Geo., - - - - - - - - - - 143
Bibliographical Notices, - - 45, 147, 345, 390, 441, 442
Atlas to the Pathology of the Teeth, - - 45, 147, 390
American Agriculturist, - - - - - - - 441
American Naturalist, - - - - - - - 441
A Treatise on Diseases of the Mouth, Jaws and Associate Parts, By Jas. E. Garretson, M.D., D.D.S., 345
Half Yearly Compendium of Medical Science, - - 147
Hitchcock's Magazine, - - - - - - - 442
Littell's Living Age, - - - - - - - - 442
Manufacturer and Builder, - - - - - - 441
Once a Month, - - - - - - - - - 442
Proceedings of Michigah State Med. Society, - 147
Scientific American, - - - - - - - 441
The Cell Doctrine. By Jas. Tyson, M.D., - - 596
The Life of the Trichina, - - - - - - 45
The New Electic Magazine, - - - - - - - 442
The Southern Review, - - - - - - - 442
Bi-Sulphide of Carbon as a Remedy for Headache, - - 98
Biting the Snake to Preserve the Teeth, - - - - 293
Bromal and Bromiform, - - - - - - - - - 487
Bryant, Geo. S., M.D., - - - - - - - - 141
Burkholder, N. M., D.D.S., - - - - - - - 209-262
Black, G. V., - - - - - - - - - - - 329-369
Blackened Teeth from Tea, - - - - - - - - 595
Bruce, W., D.D.S., - - - - - - - - - - 307-351
Calcified Pulp of a Superior Molar Tooth, By Jas. B. Hodgkin, D.D.S., - - - - - 112
Carbolic Acid, - - - - - - - - - - - 591
Carbolic Acid. Antidote to, - - - - - - - - 147
Carbolic Acid Solution. Strength of, - - - - - 146

Carbolic Glycerine and Plaster, - - - - - - - - 537
Case of Destruction of Right Half of Upper and Lower Lips and Angle of Mouth, with closure of Jaws by Cicatricial Bands, &c., By Gurdon Buck, M.D., - - 322
Carruthers, W. S., D.D.S., - - - - - - - - - 316
Case of Fibroid Tumor of Upper Jaw, By Dr. Ashurst, - 483
Change of Chemical Nomenclature, - - - - - - 267
Changes in Faculty of Baltimore College of Dental Surgery, 350
Chase, Professor H. S., M.D., D.D.S., - - - - - 38. 574
Chloral, - - - - - - - - - - - - - 387
Charleston S. C., Dental Association, - - - - - 99
Chittenden, G. S., - - - - - - - - - - 181-384
Chloroform in Infantile Convulsions, - - - - - - 595
Chromic Acid, - - - - - - - - - - - 149-294
Clasped Plates, - - - - - - - - - - - - 138
Clastic Anatomical Models. Review of Prof. F. G. Lemercier's Lecture on, By Prof. Henry R. Noel, M.D., - - 1. 51
Cleft of the Hard Palate. Surgical Treatment of, By Wm. R. Whitehead, M.D., - - - - - - - - 117
Cobb, S. J., - - - - - - - - - - - - 566
Coffee as a Deodorizer, - - - - - - - - 198
Comminuted Fracture of Nasal Bones and Superior Maxillary, 43
Complete Disarticulation of Upper Maxillary Bones. By J. H. Salter, M.R.C.S., - - - - - - 288
Consumption of Horse Meat, - - - - - - - 197
Contour Fillings. When they are Indicated. By H. L. Sage, 285
Correction. Dr. Thos. W. Evans, - - - - - - 49
Cure of Toothache, - - - - - - - - - - 296
Cutler, Professor S. P., M.D., D.D.S., - - 10. 201. 301. 456
Danger of Giving Strong Doses of Camphor, - - - - 96
De Rosset, Professor M. J., M.D., - - - - - - 412
Death from Inhalation of Bichloride of Methylene, - - 486
Death from a Dissecting Wound, - - - - - - 439
Death from Bichloride of Methylene, - - - - - - 388
Death from Taking Chloroform into the Stomach, - - 488
Death from Hypodermic Injection, - - - - - - 96
Death from Cloroform, - - - - - - - - - 595
Delay of Sept. No. of Journal, - - - - - - 250
Dental Hygiene. By Samuel Welchens, D.D.S., - - - 183
Dental Collegiate Education - - - - - - - - 347
Dentine. By Henry S. Chase, M.D., - - - - - - 38
Dentistry in Japan, - - - - - - - - - - 44
Devitalizing the Dental Pulp, and Treatment Preparatory to Filling. By J. P. Wilson, - - - - - - - 36
Disease of the Antrum, - - - - - - - - - 67. 573
Diseases of the Jaw. By Thos. Waterman, M.D., - - 86
**Drs. Morgan & Clark, - - - - - - - - - 249**

Dr. Thos. W. Evans, - - - - - - - - - - 250
Dr. Wm. Reynolds. Letter from, - - - - - - 271
Dr. Wm. H. Hoffman. Letter from, - - - - - - 275
Dr. W. W. H. Thackston. Letter to Southern Dental Association, - - - - - - - - - - 269
Dr. J. McManus. Letter from, - - - - - - 148
East Tennessee Dental Association. By W. F. Fowler, D.D.S., - - - - - - - - - - 366
Edwards Chas. G., D.D.S., - - - - - - - - 166
Effects and Treatment of Salivary and Mucous Deposits. By Louis Augspath, D.D.S., - - - - - - 567
Eidson, A. L., M.D., - - - - - - - - - 180
Encephaloid Tumor of Antrum, Removal of Superior Maxillary. By Donald McLean, M.D., L.R.C.L., - - 177
Epileptoid Convulsions Caused by Carious Teeth, - - 488
Errata, - - - - - - - - - 100. 250. 350
Excessive Opium Eating, - - - - - - - - 491
Excessive Sweating of the Hands and Feet, - - - - 97
Excision of Inferior Dental Nerve for Neuralgia, - - 195
Exposed Pulps and Fang Filling. By Thos. J. Speck, D.D.S., 502
Extirpation of an Osseous Tumor of the Upper Jaw. Wm. R. Whitehead, M.D., - - - - - - 460
Fatal Case of Tetanus Resulting from Removal of Ten Teeth while under the Influence of Nitrous Oxide Gas. By H. K. Steele, M.D., - - - - - - - 281
Fibrin, and its Relation to the Blood and Tissues. By Prof. H. R. Noel, M.D., - - - - - - 417
Fibroid Tumor of Upper Jaw, - - - - - - - 483
Fitch G. L., - - - - - - - - - - - 583
Fowler, W. F., D.D.S., - - - - - - - - - 366
Ford, Arthur C., - - - - - - - - - - 471
Foreign Objects in the Lungs, - - - - - - - 294
Fracture of Inferior Maxillary. Treatment of, By W. G. Bullock, M.D., - - - - - - - 376
Frightful Wound of the Skull. Recovery, - - - - 150
Fungous Tumor of Fangs of a Molar Tooth, Diagnosed and Treated as a Case of Menoplania. By Geo. L. Bryant, M.D., - - - - - - - 141
Furrows on the Nails as the Result of Illness, - - - - 540
Gangrenous Stomatitis. By R. S. Anderson, M.D., - - 425
George R. K., D.D.S., - - - - - - - - - 16
Georgia State Dental Society. By Arthur C. Ford, - - 471
Germinal Matter. By Thurston Wolfe, D.D.S., - - - - 508
Gold Foil. By G. V. Black, - - - - - - - - 329. 369
Gordon, James, - - - - - - - - - - - 267
Good and Bad Teeth, - - - - - - - - - 32
Goodall's Elastic or Spring Plates, - - - - - - 50

Goodwillie, D. H., M.D., D.D.S., - - - - - - - 71
Green Line on Gum from Copper Poisoning, - - - 594
Gunn, Moses, M.D., - - - - - - - - - - 432
Hæmorrhage of the Alveola. By O. E. M. Salomon, D.D.S., 363
Harris Edward N., D.D.S., - - - - - - - 367
Hard Palate. Surgical Treatment of Cleft of,
By Wm. R. Whitehead, M.D., - - - - - 117
Hawes N. W., - - - - - - - - - - - - 585
Heavy Foil. By Henry S. Chase, D.D.S., M.D., - - 519. 574
Hints on Vulcanite Work. By Jas. B. Hodgkin, D.D.S., - 251
Hodgkin, Jas. B., D.D.S., - - - - 112. 113. 161. 251
Horne, W. G., D.D.S., - - - - - - - - 236
How to Wedge, - - - - - - - - - 290
Heath, Christopher, F.R.C.S., - - - - - - - 476
How to Remove Wax from Pins of Teeth.
By B. M. Wilkerson, D.D.S., - - - - - - 366
Howard, Professor E. Lloyd, M.D., - - - - - 402
Hydrate of Chloral as an Anæsthetic, - - - - 443
Hydrate of Chloral; its Physiological Action.
By Benj. W. Richardson, F.R.C.S., - - - - 338
Hydrocyanic Acid in Medico-Legal Cases. Notes on the
Detection of, By Prof. M. J. DeRosset, M.D. - - 412
Hyperæmia and Inflammatory Exudations of Mucous Membrane of Mouth. By O. E. M. Salomon, D.D.S., - 558
Imperfect Enamel. By S. P. Cutler, M.D., D.D.S., - - 144
Indication of Longevity, - - - - - - - 594
In Toe—Toe, - - - - - - - - - - - 320
Injunction Against Boston Dental College, - - - 150
Insanity Cured by Removal of Carious Teeth.
By W. T. Perry, M.D., - - - - - - - - 62
Interdental Splints for Fractures of Inferior Maxilla.
By G. L. Fitch, - - - - - - - - 583
Investigation of a Malformed Tooth. By O. E. M.
Salomon, D.D.S., - - - - - - - - 173
Iodal, - - - - - - - - - - - - - 487
Iodine and Carbonic Acid for Local Application, - - 198
Irregular Dentition and Caries of the Lower Jaw, - - 489
Keesee, Geo. Fiske, D.D.S., - - - - - - - 107
Large Multilocular Cystic Tumor of Lower Jaw Removed
by Excision. By W. B. Beatson, F.R.C.S., - - 276
Large Salivary Calculi, - - - - - - - - - 439
Letherby, Dr. M. A., - - - - - - - - 93. 131
Leute, Frederick D., M.D., - - - - - - - 40
Letters, - - - - - - - 25. 116. 148. 269. 271. 275
Local Anæsthesia for Incipient Alveolar Abscess, - - 99
Maclean, Donald, M.D., L.R.C.L. - - - - - - 177
Manufacturing Instruments. By A Student, - - - 420
Mercurius Vivus. By C. S. Chittenden, - - - 384

Mercury Fumes, - - - - - - - - - - 295
Mercury in India Rubber Dental Plates.
By G. H. Perine, D.D.S., - - - - - - - 576
Method of Mounting Gum Teeth on Rubber and Protecting the Joints. By J. Jordon, - - - - - - - 267
Microscopy of the Dental Tissues. By S. P. Cutler, M.D., D.D.S., A.E.G., - - - - - - - 10, 301, 456
Microscopy of the Teeth. By S. P. Cutler, M.D., A.E.G., D.D.S., 201
Morgan's Plastic Gold, - - - - - - - - 589
Muscular Contraction after Congelation, - - - - 286
Necrosis of Nearly the Whole of Lower Jaw—Removal of Dead Bone, Including one Condyle—Recovery with Perfect movement of Jaw. By Christopher Heath, F.R.C.S., - - - - - - - - - 476
Neuralgia. By Chas. G. Edwards, D.D.S., - - - 166
Neuralgia and its Treatment by Electrization, - - - 593
New Hæmostatic Collodion, - - - - - - - 297
New Mode of Administering Nitrous Oxide Gas, - - - 445
New Operation in Dental Science. By Q. L. Adams, D.D.S., 279
Noel, Professor Henry R., M.D. - - - - 1, 51, 417, 449
Notes from Dental Practice. A Peculiar Form of Ulceration of Gums, - - - - - - - - - - 24
A Singular Case, - - - - - - 113
Disease of the Antrum, - - - - - - 67
Calcified Pulp of a Superior Molar, - - 112
Treatment of Exposed Pulps, - - - 22
On the Necessity of Artistic Knowledge and Critical Taste to Highest Success in the Dental Profession. By G. H. Perine, D.D.S., - - - - - - 26
Osseous Growths in the Gums. By Thurston Wolfe, D.D.S., 63
Painless Cutting in Surgery, - - - - - - - 440
Paradoxes in Dentistry. By Professor Austen, - - - 399
Pathological Specimens of Teeth, - - - - - - 495
Patterson, J. D. - - - - - - - - - - 66
Periodontitis. By J. F. Babcock, - - - - - - 473
Per—Manganate of Potash, - - - - - - - 350
Perine, G. H., D.D.S., - - - - - - - 26, 576
Perry, W. T., M.D., - - - - - - - - - 62
Pivot Teeth. By James B. Bean, D.D.S., M.D., - - 151
Pivot Teeth. By J. Hardman, - - - - - - - 381
Plaster as an Impression Material. By R. K. George, D.D.S., 16
Plaster Impressions, - - - - - - - - - 44
Poisoning by Tincture of Aconite, - - - - - - 544
Position of the Body Favoring Sleep, - - - - - - 295
Prang's Chromos, - - - - - - - - - 496
Prussic Acid, - - - - - - - - - - 298
Recent Histological Views as Regards Enamel and Dentine. By Prof. H. R. Noel, M. D. - - - - - 449

Redman, Prof. W. G. - - - - - - - - - 300
Remedy for Carious Teeth. - - - - - - - - 299
Removal of One-Half of Inferior Maxillary for Osteo-Sarcoma.
By S. R. Beckwith, M. D. - - - - - - 283
Replantation, Transplantation and Implantation.
By O. E. M. Salomon, D. D. S. - - - - - 113
Restoration of a Portion of the Jaw. By J. D. Patterson, - 66
Review of Prof. F. G. Lemercier's Lecture.
By Prof. H. R. Noel, M. D. - - - - - - 1, 51
Richardson, Benj. W. - - - - - - - - 338
Rhoades, Sumner M. D. - - - - - - - - - 434
Salivary Calculus from Wharton's Duct. - - - - 198
Salomon, O. E. M. D.D.S. - - - - - - 173, 363, 558
Salter, J. H. M.R.C.S., L. S. A. &c., - - - - 288
Salutatory, - - - - - - - - - 46
Scarification of the Gums in First Dentition, - - - 390
Skin Diseases, - - - - - - - - - - 542
Sleeping Sickness, (Maladie du Sommeil.) - - - - 98
Smith, G. Stevenson L. R. C. S. E. - - - - - - 579
Solutions of Protroxide of Nitrogen, - - - - 538
Some Unusual Phenomena attending Anæthesia.
By Fred. D. Leute, M. D. - - - - - - 40
Something New in the Working of Plaster of Paris, - - 430
Soothing Syrup, - - - - - - - - - 439
South Carolina Dental Association, - - - - - - 601
Southern Dental Association, - - - - - - 47
Southern Dental Association. Second Annual Meeting of - 546
Southern Dental Association. Proceeding of First Annual
Meeting of By. Jno. G. Angill, D.D.S. - - - 217
Special Dental Journals, - - - - - - - - 596
Spectral Analysis, - - - - - - - - - 469
Staphyloraphy. By Moses Gunn, M. D. - - - - 432
Strange Monstrosity, - - - - - - - - 293
Strength of Carbolic Acid Solutions, - - - - 146
Styptic Paper, - - - - - - - - - - 293
Speck, Thos. J., D.D.S. - - - - - - - - 502
Substances Eliminated in the Breath, - - - - - 489
Sulpho-Vinic Acid, - - - - - - - - - 298
Surgical Treatment of Cleft of Hard Palate. By Wm. R.
Whitehead, M. D. - - - - - - - - - 117
Suspension of New York College of Dentistry, - - - - 100
Sweet Quinine, - - - - - - - - - - 294
Teething Syrups and Powders, - - - - - - 495
Tennessee State Dental Association, - - - - - 71
Tetanus, - - - - - - - - - - - - 386
Tetanus. To Prevent, - - - - - - - - 542
The Artificial Leech, - - - - - - - - 487
The Blood. By Geo. Fiske Keesee, D.D.S., - - - 107

The Circulation. By N. M. Burkholder, D.D.S., - - 209, 262
The Cause of the Suspension of the New York College of Dentistry, - - - - - - - - - 199
The Fifth Pair of Nerves. By W. S. Carruthers, D.D.S., 316
The Hygrometer, - - - - - - - - 516
The Inferior Maxilla and its Changes. By John R. Barr, D.D.S. 256
The Injunction Case of the Boston Dental College, - - 200
The Mallet vs. Hand Pressure, - - - - - - - - 148
The New York College of Dentistry, - - - - - - - 496
The Meeting of the American Dental Association, - - 100
The Organization of the Southern Dental Association, - - 248
The Perils of Fashion, - - - - - - - - - - 536
The Inter-dependence of Diseases of the Teeth and of the Female Pelvic Organs. By N. W. Hawes, - - 585
The Use of an Amalgam of Mercury and other Metals in Filling Teeth, - - - - - - - - 491
The Three Varieties of Dyspepsia, - - - - - 97
Three New Anæsthetics—Iodal, Bromal, and Bromiform, - 487
Tobacco—The Quantity Consumed and its Effects on Youth, 389
To Cool Water, - - - - - - - - - - - 344
To Prevent Death by Chloroform, - - - - - - 197
Toothache Among the Ancients, - - - - - - 490
Treatment of Alveolar Abscess. By C. S. Chittenden, - 181
Treatment of Diarrhœa, - - - - - - - - - 196
Treatment of Fracture of Inferior Maxillary, by an Improved Apparatus. By W. G. Bullock, M. D. - - - - 376
Treatment of Tooth Pulps. By J. G. Willis, - - 478
Two Cases of Convulsions Arrested by Scarification of the Gums. By G. S. Smith, L. R. C. S. E., - - - 579
Use of Gum-Cotton in Dentistry, - - - - - - - 296
Varieties of Food—their Chemical Composition and Nutritive Value. By Dr. Letherby, M. A. - - - - - 93, 131
Vulcanite. By Professor Austen, - - - - - 497
Vulcanite Work. Hints on By Jas. B. Hodgkin, D.D.S. 251
Warts, - - - - - - - - - - - - - 446
Waterman, Thos. M. D. - - - - - - - - 86
Welchens, Saml. D.D.S., - - - - - - - - 183
Whitehead, Wm. R. M.D. - - - - - - 117, 460
Weston's Metals, - - - - - - - - - - - 600
White's Dentifries, &c., - - - - - - - - 50
Why do we Oil our Whetstones, - - - - - - 541
Why is the Production of Pus a Cause of Exhaustion, - 388
Wilkerson, B. M., D.D.S. - - - - - - - - 366
Willis, J. G. - - - - - - - - - - - - 478
Wilson, J. P. - - - - - - - - - - - 36
Wolfe, Thruston D.D.S. - - - - - - - 63, 508
Westcott, A. M.D. D.D.S., - - - - - - - - 523

### TO READERS AND CORRESPONDENTS.

Communications intended for publication, and exchange journals and papers, should be addressed to the EDITOR OF THE AMERICAN JOURNAL OF DENTAL SCIENCE, 259 N. Eutaw St., Baltimore, Md. All letters relating to the business of the journal, or containing cash remittances, to be directed to Messrs. SNOWDEN & COWMAN. Articles for publication should be received by the editor at least three weeks previous to the first day of the month on which the number in which it is designed for them to appear, is issued.

### CONTENTS:

### ORIGINAL COMMUNICATIONS.

### MONTHLY SUMMARY.

Comminuted Fracture of Nasal Bones and Superior Maxilla; Sinking of Eyeball into Maxillary Sinus .......... 43

Dentistry in Japan .......... 44

Plaster Impressions .......... 44

### BIBLIOGRAPHICAL NOTICES.

Atlas to the Pathology of the Teeth .......... 45
The Life of the Trichina .......... 45

### EDITORIAL DEPARTMENT.

Salutatory .......... 46

Southern Dental Association .......... 47

Correction. Dr. Thos. W. Evans .......... 49

White's Dentifrices, &c .......... 50

Goodall's Elastic or Spring Plates. .......... 50

☞ The American Journal of Dental Science will contain fifty pages of reading matter in each number, making a volume of not less than *Six Hundred* pages;

EXCLUSIVE OF ADVERTISEMENTS.

### PROSPECTUS

### Of the 3d. Volume of the Third Series

OF THE

### AMERICAN JOURNAL

OF

### DENTAL SCIENCE.

F. J. S. GORGAS, M. D., D. D. S., Editor.

---

The MAY number commences a new volume of this *Journal*, the oldest periodical in the world devoted to the propagation of the principles upon which Dental Surgery is founded. It is issued on the first of each month and contains not less than *fifty* pages of reading matter in each number, exclusive of advertisements, making a volume of *six hundred* pages per annum.

In each number will be found Original Communications, Correspondence, Selected Articles, Monthly Summary, Bibliographical Notices and Editorial Matter, and every effort will be exerted to render the *Journal* worthy of the patronage of the Dental profession.

The object of the editor in undertaking the duties and responsibilities devolving upon him is to make the *Journal* an exponent of the status of dentistry, and a medium by which members of the dental profession can communicate their ideas to their fellows, and thus diffuse information and cultivate the habit of imparting it. The latest improvements will be duly noticed and all inventions and suggestions of any merit will be fully described and impartially discussed, and every honest effort made to advance the best interests of the profession.

A generous co-operation is respectfully solicited from the members of the profession; and as the *Journal* has now a printing office of its own which materially lessens the cost of its publication, it will be issued at the reduced subscription price of $2.50 per annum in advance.

Communications are respectfully solicited on all subjects pertaining to the practice of dentistry, as well as reports of cases occurring in dental practice.

All original communications designed for publication, works for review, new instruments for notice, exchanges, &c., should be directed to the editor, 259 N. Eutaw St. Baltimore, Md.

All advertisements and subscriptions should be addressed to

SNOWDEN & COWMAN,

*Publishers of Am. Journal of Dental Science.*

86 W. FAYETTE ST. BET. CHARLES & LIBERTY STS.,

BALTIMORE, Md.

